# Antioxidant Capacity of Pure Compounds and Complex Mixtures Evaluated by the ORAC-Pyrogallol Red Assay in the Presence of Triton X-100 Micelles 

**DOI:** 10.3390/molecules15096152

**Published:** 2010-09-01

**Authors:** Max Romero, Benjamin Rojano, Jaime Mella-Raipán, Carlos David Pessoa-Mahana, Eduardo Lissi, Camilo López-Alarcón

**Affiliations:** 1 Departamento de Farmacia, Facultad de Química, Pontificia Universidad Católica de Chile; C.P. 782 0436, Santiago, Chile; E-Mails: msromero@uc.cl (M.R.); predestinar@gmail.com (J.M.); cpessoa@uc.cl (C.D.P.M.); 2 Escuela de Química, Universidad Nacional de Colombia, Sede Medellin; AA 1027 Medellin, Colombia; E-Mail: brojano@gmail.com; 3 Facultad de Química y Biología, Universidad de Santiago de Chile, Santiago, Chile; E-Mail: eduardo.lissi@usach.cl

**Keywords:** ORAC, pyrogallol red, micelles, Triton X-100, antioxidants

## Abstract

The protective effect of different antioxidants and complex mixtures on the consumption of pyrogallol red (PGR) induced by peroxyl radicals was studied in the absence and presence of Triton X-100 micelles. The presence of micelles decreased significantly the protection of PGR afforded by lipophilic antioxidants (β-carotene, octyl gallate), while no effect of micelles was observed for hydrophilic antioxidants such as Trolox, caffeic acid, gallic acid, and ascorbic acid. In the presence of complex mixtures a clear effect of Triton X-100 micelles was also observed in the protection afforded by wines, tea infusions, and seed extracts of *Eugenia jambolana* and *Myrciaria cauliflora*. On the other hand, no effect of micelles was observed for orange juice and pulp fruit extracts. The ORAC (Oxygen Radical Absorbance Capacity) index was evaluated in the absence (ORAC-PGR) and presence of Triton X-100 micelles (ORAC-PGR_MIC_). Triton X-100 micelles affect ORAC-PGR values of antioxidants in a lipophilicity-dependent way. From the obtained results, we conclude that ORAC-PGR and ORAC-PGR_MIC_ assays could be considered as an alternative to estimate the antioxidant ability (ORAC-PGR) and to infer the association to Triton X-100 micelles (ORAC-PGR/ORAC-PGR_MIC_) of pure antioxidants and their complex mixtures.

## 1. Introduction

It is recognized that reactive oxygen specie (ROS) are implicated in all stages of lipid peroxidation (initiation, propagation, and termination) [1]. Depending of the nature of the lipid, these oxidative reactions could modify oil composition, cell membranes, or the integrity of lipoproteins [1,2,3]. There is abundant evidence that polyphenols present in foods and beverages can inhibit lipid peroxidation processes acting as chain breaking antioxidants [1,4], being the efficiency of a particular antioxidant mainly determined by:

- its reactivity towards ROS,
- the stoichiometry of the reaction (number of ROS molecules that an antioxidant molecule can remove),
- its ability to reach lipidic phases, and
- its capacity to generate, after its reaction with ROS, products with low reactivity towards lipids.

There is increasing interest concerning the measurement of the antioxidant capacity of polyphenols such as flavonoids, and related compounds. Several methodologies have been developed to allow these evaluations, with the ORAC (Oxygen Radical Absorbance Capacity) assay being one of the most frequently employed. This method is based on the use of a target molecule that is exposed to a peroxyl radical source in the absence and presence of the tested sample. Usually fluorescein (ORAC-FL), and 2,2’-azo-bis(2-amidinopropane) dihydrochloride (AAPH) are employed as probe and peroxyl radical source, respectively [5,6]. To evaluate the antioxidant capacity of lipophilic antioxidants, the ORAC-FL assay has been validated employing randomly methylated β-cyclodextrin (RMC) as solubilizating agent (L-ORAC-FL) [7,8,9]. The consumption of fluorescein, either in ORAC-FL or L-ORAC-FL assays, usually is easily inhibited by single antioxidants and/or complex mixtures including very reactive compounds, generating kinetics profiles characterized by the presence of neat induction times. This would imply that ORAC values evaluated employing fluorescein as probe would be more related to stoichiometric factors than to the reactivity of the antioxidants. In this context, we have previously proposed that ORAC values obtained employing pyrogallol red (PGR) as probe (ORAC-PGR) would be more related to the reactivity of the additives toward peroxyl radicals than to the stoichiometry of the reaction [10,11]. In fact, in contrast with fluorescein, the kinetic profiles of the PGR consumption do not present induction times, even in the presence of very reactive polyphenols. In particular, ascorbic acid was the only antioxidant, among the tested compounds, that generated a clear induction time [11,12]. The ORAC-PGR method has been used, as a complementary methodology of ORAC-FL, to estimate the antioxidant capacity of complex mixtures including beverages [13,14,15], wines [10,11], fermented foods [16] fruit extracts [12,17], and human fluids [18,19]. 

Taking into account the relevance of the hydrophilicity/lipophilicity balance of antioxidants in the inhibition of lipid peroxidation processes, and the advantages of the use of PGR as probe in ORAC-like assays, in the present work we evaluated the ORAC-PGR index of hydrophilic and lipophilic antioxidants in Triton X-100 micelles. For this purpose, antioxidants with different degree of hydro/lipophilicity were used. In addition, the antioxidant capacity of complex mixtures such as tea infusions, berry extracts, wines, and orange juice was evaluated.

## 2. Kinetic Scheme

**Scheme 1:**
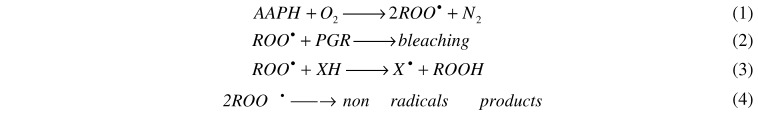


ORAC-PGR methodology has been used to evaluate the antioxidant capacity of antioxidants, beverages, fruit extracts, and human fluids in aqueous homogeneus medium [10,11,12,13,14]. The minimal set of reactions to consider in the ORAC-PGR assay is given in Scheme 1, where XH stands for the added antioxidant(s).

**Scheme 2:**
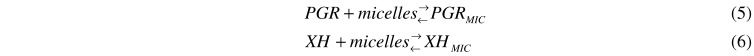


For simplicity self-reactions and cross-reactions of the radicals produced in steps (2) and (3), and the formation of alcoxyl radicals in reaction (4), are not included. The addition of Triton X-100 micelles to a solution containing AAPH, PGR and an antioxidant (or a complex mixture) could modify the rate of the reactions depicted in Scheme 1. Since all processes could take place in the external phase and/or in the micellar pseudophase, the relevance of these processes is going to be determined by the distribution of the compounds between the aqueous phase (phosphate buffer) and the less polar micellar pseudophase. Then, in presence of Triton X-100 micelles, besides the reactions of Scheme 1 the equilibria presented in Scheme 2 should also be considered.

Considering the hydrophilic character and the positive charge (in phosphate buffer) of AAPH [6], it can be assumed that all peroxyl radicals are generated (and remain) in the phosphate buffer pseudo-phase. Therefore, the reaction of PGR and the antioxidants with peroxyl radicals would take place in the aqueous phase and/or the interface micelle-phosphate buffer. 

## 3. Results and Discussion

### 3.1. Consumption of PGR Induced by Peroxyl Radicals in Presence of Triton X-100 Micelles

**Figure 1:**
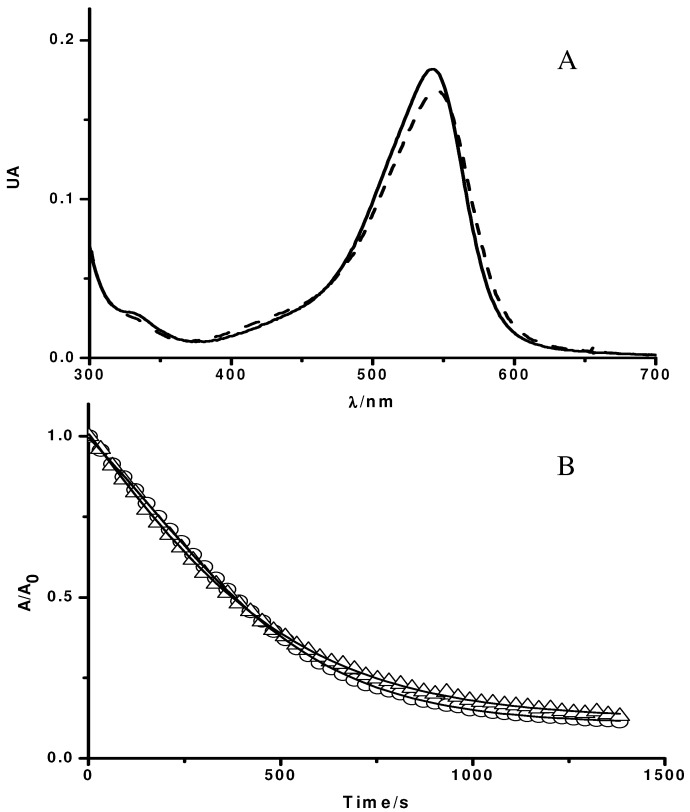
Influence of Triton X-100 micelles on the UV-visible spectrum of PGR (**A**) and its consumption mediated by peroxyl radicals (**B**). **A**: UV-visible spectrum of PGR (5 µM) in phosphate buffer (75 mM, pH 7.4, solid line), and in the presence of Triton X-100 (50 mM) (dashed line); **B**: Time-course of the consumption of PGR induced by AAPH derived peroxyl radicals in the absence and presence of Triton X-100 micelles. PGR (5 µM) was incubated in phosphate buffer (75 mM, pH 7.4) at 37 °C with AAPH (10 mM) in the absence (○), and presence of Triton X-100 (50 mM, △).

Figure 1A shows UV-visible spectra of PGR in the absence and presence of Triton X-100 micelles (50 mM). As can be seen, in the absence of Triton X-100 micelles, PGR presented the typical UV-visible spectrum (solid line in Figure 1), characterized by a band in the visible region with a maximum at 540 nm (ε = 36,000 M^-1^cm^-1^). In the presence of Triton X-100 micelles, the spectrum of PGR was slightly altered (dashed line in Figure 1), displacing the maximum of the visible band to 544 nm (ε = 33600 M^-1^cm^-1^). This behavior would imply that PGR was, at least partially, associated to the micellar pseudophase. In fact, by an ultrafiltration separation process it was observed that, in our experimental conditions (Triton X-100 50 mM, and PGR 5 µM), near 72% of PGR would be associated to Triton X-100 micelles. Nevertheless, the association of PGR to Triton X-100 micelles did not modify the kinetic profile of PGR consumption induced by peroxyl radicals. As is shown in Figure 1B, the consumption of PGR mediated by AAPH derived peroxyl radicals (evaluated by its absorbance decay at 540 nm) was not affected by the presence of Triton X-100 micelles. This is an unexpected result if it is considered that AAPH and its derived free radicals are mostly in the aqueous phase, while a significant fraction of PGR is associated to the micelles. However, the PGR/peroxyl radical process (Reaction 2, Scheme 1) is in the zero order kinetic limit [10,11] and hence its consumption rate is independent of the PGR concentration present in the aqueous phase. In fact, similar initial rate values (≈ 0.4 µM/min) of PGR decay were estimated in the absence and presence of Triton X-100 micelles. Considering that peroxyl radicals are generated (APPH = 10 mM, at 37 °C) at a rate of 0.8 µM/min [6], and that each PGR molecule reacts with two peroxyl radicals [11], the estimated rates of PGR consumption in the absence and presence of Triton X-100 micelles supports the proposal that PGR is in the zero order kinetic limit. Furthermore, PGR adsorbed at the micelles interface could retain its reactivity toward the peroxyl radicals.

### 3.2. Effect of Single Antioxidants on the PGR Consumption in Presence of Triton X-100 Micelles

**Figure 2:**
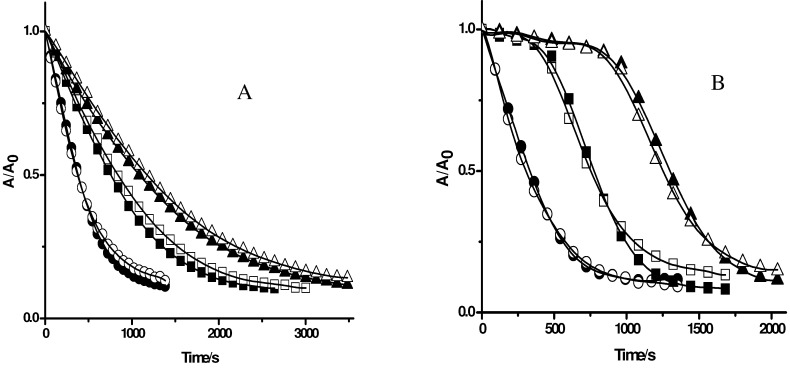
Effect of antioxidants on the consumption of PGR induced by AAPH derived peroxyl radicals in the absence (closed symbols) and presence of Triton X-100 micelles (open symbols). PGR (5 µM) was incubated with AAPH (10 mM) in the absence (circles) and presence of. **A**: Trolox: 50 (squares), and 100 µM (triangles); **B**: Ascorbic acid: 5 (squares), and 10 µM (triangles).

**Figure 3:**
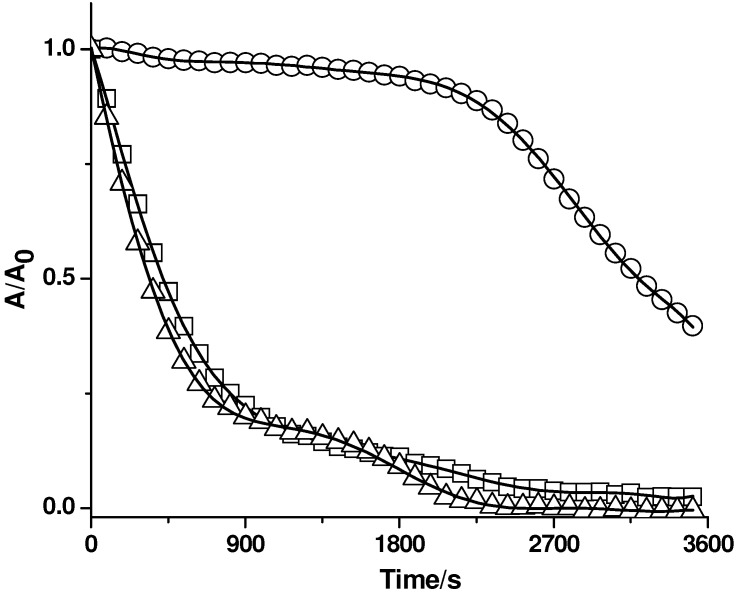
Effect of β-carotene on the consumption of PGR induced by AAPH derived peroxyl radicals. PGR (5 µM) was incubated with AAPH (10 mM) and Triton X-100 (50 mM) in the absence and presence of β-carotene (5 µM). The reaction was followed by the consumption of PGR (□, 550 nm) and β-carotene (○, 460 nm). Control experiment without β-carotene (△).

To evaluate the effect of Triton X-100 micelles on the protection of PGR afforded by pure compounds, different antioxidants with different hydro/lipophilicity were employed. Figure 2 shows the consumption of PGR (5 µM) induced by AAPH derived peroxyl radicals in the presence of Trolox, and ascorbic acid with or without Triton X-100 micelles. As is shown in Figure 2A, Trolox (50 and 100 µM) inhibited the consumption of PGR in a concentration-dependent way, showing a similar behaviour to that observed in previous studies in aqueous media [10,11]. Interestingly, the addition of Triton X-100 micelles did not modify the protection of PGR afforded by Trolox, a result that would be related to the well known hydrophilic property of this antioxidant. In the same way, the addition of Triton X-100 micelles did not alter the protective effect afforded by ascorbic acid, characterized by a neat induction time (Figure 2B). These similarities can be attributed to the fact that, even in presence of Triton X-100 micelles, all relevant processes take place almost exclusively in the aqueous phase. In contrast with this, when a lipophilic antioxidant as β-carotene was added to a solution containing Triton X-100 micelles, PGR and AAPH, no PGR protection by the antioxidant took place (Figure 3). In fact, as it is shown in Figure 3, the absorption band corresponding to β-carotene (at 460 nm) was not affected by peroxyl radicals during the consumption of PGR. Nonetheless, when PGR was almost totally consumed (c.a. 37 minutes), the visible band (at 460 nm) associated to β-carotene, decayed significantly. These results imply that β-carotene (present in the micelles) was initially protected by PGR (present in the aqueous phase and/or in the micelle-aqueous interface), and only after the total consumption of PGR, β-carotene was consumed by peroxyl radicals (probably in the interface micelle-phosphate buffer). Similarly, β-carotene was efficiently protected by Trolox and ascorbic acid (data not shown), implying that hydrophilic antioxidants react with peroxyl radicals in the aqueous phase, minimizing its interaction with micelle associated targets (as β-carotene). Control experiments with β-carotene micellar solutions revealed no changes in the original absorbance intensity at 460 nm (data not shown) in the time period considered in the presence work.

**Figure 4:**
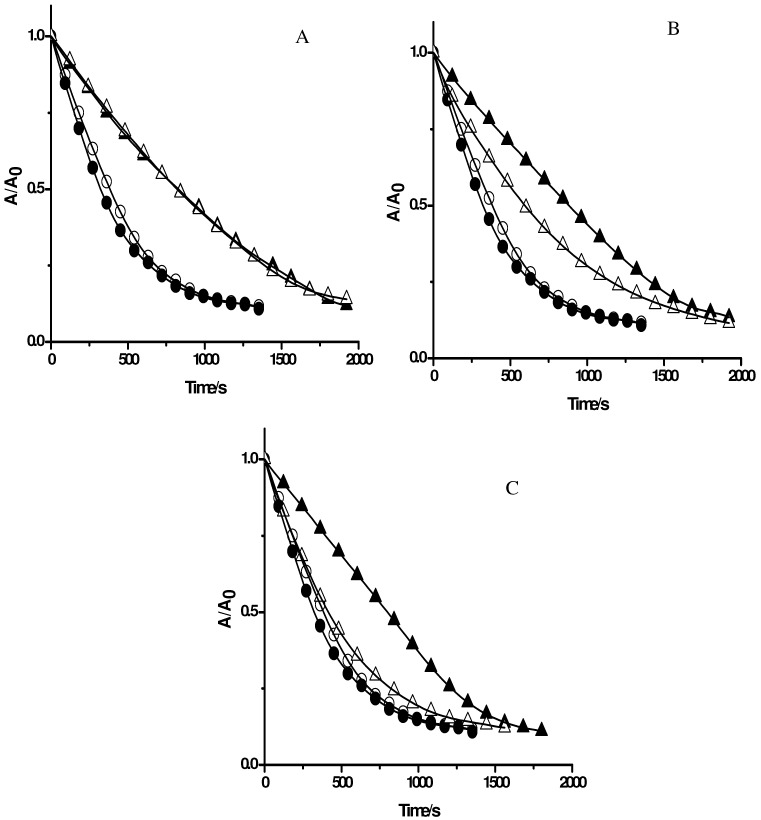
Effect of gallate derivatives (**A** gallic acid; **B** propyl gallate; **C** octyl gallate) on the consumption of PGR induced by AAPH derived peroxyl radicals in the absence and presence of Triton X-100 micelles. PGR (5 µM) was incubated with AAPH (10 mM) and the gallate derivatives (at 5 µM) in micellar (△) and phosphate solutions (▲). Control experiments without gallate derivatives were carried out in the presence (○) and absence (●) of Triton X-100 micelles.

**Figure 5:**
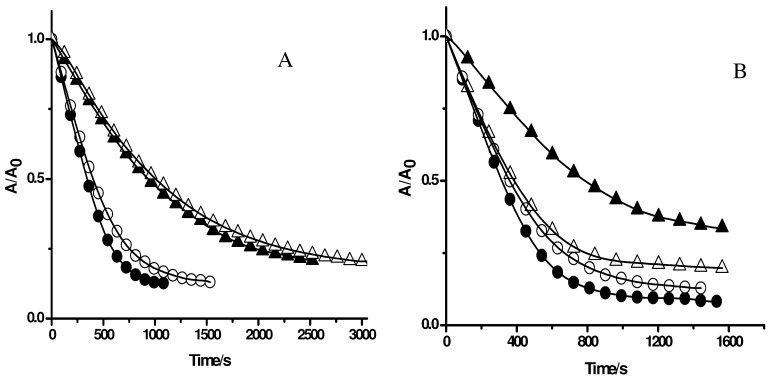
Effect of caffeic acid (**A**) and ethyl caffeate (**B**) on the consumption of PGR induced by AAPH derived peroxyl radicals in the absence and presence of Triton X-100 micelles. PGR (5 µM) was incubated with AAPH (10 mM) and the caffeic acid derivatives (at 250 µM) in micellar (△) and phosphate solutions (▲). Control experiments without caffeic acid derivatives were carried out in the presence (○) and absence (●) of Triton X-100 micelles.

**Figure 6:**
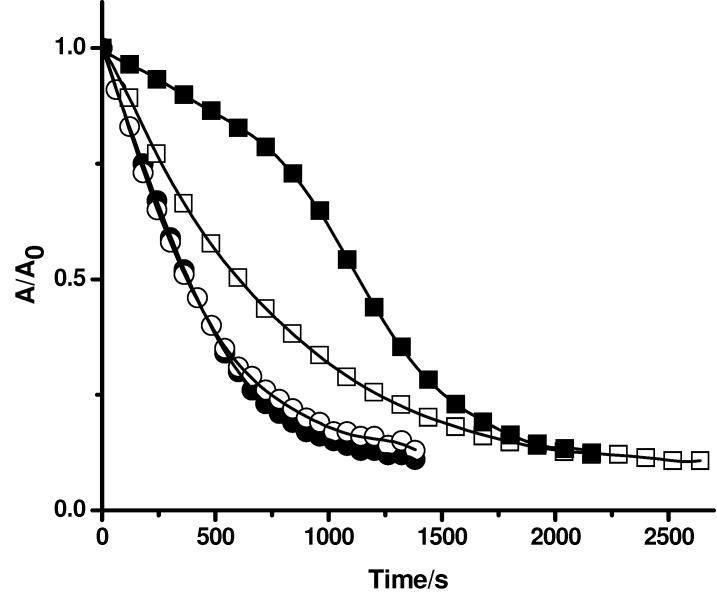
Effect of quercetin (7 µM) on the consumption of PGR (5 µM) induced by AAPH derived peroxyl radicals in the absence (■) and presence of Triton X-100 micelles (□). Control experiments without quercetin were carried out in the presence (○) and absence (●) of Triton X-100 micelles.

To evaluate the influence of Triton X-100 micelles on the protection of PGR afforded by others antioxidants, compounds with a similar reactive centre, but with different degree of lipophilicity, were used. For this purpose, gallic acid and gallate derivatives with different alkyl chain lengths, such as methyl gallate, propyl gallate, and octyl gallate, were employed as model antioxidants. As can be seen in Figure 4A, the consumption of PGR in the presence of gallic acid at 5 µM concentration was very similar in the absence and presence of Triton X-100 micelles. This result is compatible with those obtained employing Trolox and ascorbic acid (Figure 2). However, in the case of propyl, and octyl gallate (Figure 4B, and 4C, respectively) a clear effect of Triton X-100 micelles was observed. In the presence of Triton X-100 micelles the protection afforded by propyl gallate was lower than that the observed in buffer. This would imply that the incorporation of propyl gallate into the micellar pseudophase leads to a lower availability of propyl gallate to react with peroxyl radicals. An extreme case was observed for octyl gallate. The presence of Triton X-100 micelles significantly decreased the observed protection of PGR afforded by this antioxidant in homogeneous medium, indicating that octyl gallate was mostly incorporated in Triton X-100 micelles (Figure 4C). This corroborates the results obtained with β-carotene (Figure 3) indicating that the free radical scavengers incorporated into the micelles do not protect PGR from its consumption mediated by peroxyl radicals. Likewise, we assessed the effect of caffeic acid and ethyl caffeate on the consumption of PGR induced by AAPH derived peroxyl radicals. As shown in Figure 5A, the protective effect of caffeic acid on the consumption of PGR was independent of the presence of Triton X-100 micelles. However, the protection of PGR afforded by ethyl caffeate decreased significantly in the micellar solution (Figure 5B). A similar behaviour was observed for quercetin (Figure 6). Quercetin, in phosphate buffer protected efficiently PGR, while in presence of Triton X-100 micelles, its protective effect decreased considerably.

**Figure 7:**
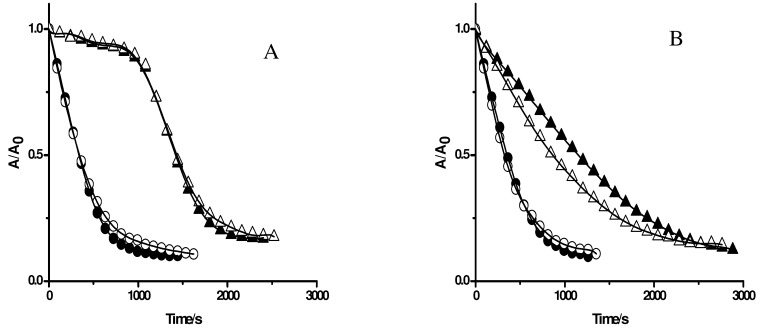
Effect of complex mixtures on the consumption of PGR (5 µM) induced by AAPH (10 mM) derived peroxyl radicals. **A**: orange juice (6.7 µL/mL) in the absence (▲) and presence of Triton X-100 micelles (△). **B**: seed extract of *Eugenia jambolana* (10 µL/mL) in the absence (▲) and presence of Triton X-100 micelles (△). Control experiments (without additives) were measured in the absence (●) and presence (○) of Triton X-100 micelles.

The above results imply that the protection of PGR is strongly influenced by the distribution of the antioxidants in Triton X-100 micelles. In particular, protection by water soluble substrates is similar to that measured in the absence of Triton X-100 micelles. On the other hand, highly hydrophobic substrates do not protect PGR in the presence of Triton X-100 micelles. Antioxidants that partition between both pseudophases (such as quercetin) present an intermediate behaviour. Thus, in the presence of complex mixtures containing antioxidants, the observed effect of Triton X-100 micelles on the kinetic profiles of PGR consumption could be directly related to the percentage of lipophilic antioxidants included in the sample. To study the latter, we estimated the inhibition of PGR consumption induced by AAPH derived peroxyl radicals by complex mixtures such as wines, juices, berry extracts, and tea infusions in the absence and presence of Triton X-100 micelles. Figure 7 shows the protection of PGR afforded by orange juice (Figure 7A) and a seed extract of *Eugenia jambolana* (Figure 7B). As can be seen in this figure, orange juice protected PGR throughout a clear induction time. This induction time, originated by the presence of ascorbic acid, was not altered by the presence of Triton X-100 micelles. This result is in accord to those obtained regarding the antioxidant capacity of pure ascorbic acid and implies that the protection of orange juice is almost exclusively related to this antioxidant. Seed extracts of *Eugenia jambolana* inhibited efficiently the consumption of PGR induced by peroxyl radicals (Figure 7B). However, in contrast to orange juice, the effect of the seed extract was dependent of the presence of Triton X-100 micelles. Thus, in the presence of micelles, a lower effect was registered, implying that lipophilic antioxidants contribute to the antioxidant capacity of this extract.

### 3.3. ORAC Values of Single Antioxidants and Complex Mixtures in the Absence and Presence of Triton X-100 Micelles

In order to quantify the effect of Triton X-100 micelles, ORAC-PGR values of pure antioxidants and complex mixtures were estimated in the absence (ORAC-PGR) and presence of Triton X-100 micelles (ORAC-PGR_MIC_). Gallate derivatives showed similar ORAC-PGR values, being 11.7, and 9.8 the ORAC-PGR value of gallic acid, and octyl gallate, respectively (Table 1). The similarity of ORAC-PGR values of gallic acid and gallate derivatives of different hydrophobicity can be related to their common reactive centre. ORAC-PGR_MIC _values of the gallate derivatives are also given in Table 1. It is observed an inverse dependence with the chain length. Thus, ORAC-PGR_MIC _index of the gallate derivatives follows the order: Gallic acid > methyl gallate > propyl gallate > octyl gallate.

**Table 1:** ORAC-PGR and ORAC-PGR_MIC_ values of pure antioxidants.

Compound	ORAC-PGR	ORAC-PGR_MIC_	ORAC-PGR/ORAC-PGR_MIC_
Gallic acid	11.7± 0.5	10.9 ± 0.4	1.1 ± 0.1
Methyl gallate	12.0 ± 0.4	7.0 ± 0.6	1.7 ± 0.2
Propyl gallate	12.3 ± 0.2	6.3 ± 0.5	1.9 ± 0.2
Octyl gallate	9.8 ± 0.3	1.6 ± 0.2	6.1 ± 0.9
Caffeic acid	0.4 ± 0.05	0.4 ± 0.07	1.0 ± 0.3
Ethyl caffeate	0.4 ± 0.06	0.2 ± 0.01	2.0 ± 0.4
Quercetin	11.9 ± 0.4	4.1 ± 0.2	2.9 ± 0.2
Ascorbic acid	10.3 ± 0.3	10.7 ± 0.5	1.0 ± 0.08
β-carotene	----	≈ 0.1	---
Trolox	1	1	1

This result can be related to the lipophilicity of gallate derivatives and hence is related to their distribution upon Triton X-100 micelles. Thus, ORAC-PGR_MIC_ values are inversely related to the distribution of the antioxidants in Triton X-100 micelles. In fact, the reported values for the partition constant of gallate derivatives between Triton X-100 micelles and aqueous solutions range from 97 to 440 M^-1^ for methyl and octyl gallate, respectively [20]. A similar effect of Triton X-100 micelles was observed for the couple caffeic acid / ethyl caffeate (Table 1). While the ORAC-PGR value of caffeic acid was not altered by Triton X-100 micelles (0.4 in both media), ORAC-PGR_MIC_ of ethyl caffeate was only one half of its ORAC-PGR value (ORAC-PGR_MIC_ = 0.2). In addition, ORAC-PGR and ORAC-PGR_MIC_ values of quercetin and ascorbic acid showed that while the antioxidant capacity of quercetin decreased significantly in presence of Triton X-100 micelles, no effect of micelles was observed for the ascorbic acid antioxidant ability. These results imply that the comparison of ORAC-PGR and ORAC-PGR_MIC_ values reflects the distribution of the antioxidant in Triton X-100 micelles. Thus, the ratio ORAC-PGR/ORAC-PGR_MIC_ can be considered as an index related to the lipophilicity of the tested antioxidant(s). As shown in Table 1, ORAC-PGR/ORAC-PGR_MIC_ values of gallate derivatives between 1.0 and 6.1 for gallic acid and octyl gallate, respectively, were obtained. In addition, ORAC-PGR/ORAC-PGR_MIC _values higher than one were obtained for antioxidants such as ethyl caffeate, and quercetin, compounds that can be considered to partition between the micelles and the dispersium solvent. 

The obtained results (collected in Table 1) employing single antioxidants suggest that ORAC-PGR and ORAC-PGR_MIC_ assays could be used to estimate the antioxidant capacity and the distribution of antioxidants in Triton X-100 micelles of complex mixtures such as wines, tea infusion, fruit extracts, and juices. As can be seen in Table 2, ORAC-PGR values of these mixtures ranged from 2 to 36 mM Trolox equivalents, values that are related to the antioxidant activity of the samples. These ORAC-PGR values were influenced in different grades by the presence of Triton X-100 micelles. In fact, ORAC-PGR_MIC_ values of wines, tea infusions, and seed fruit extracts, were lower than ORAC-PGR values, implying that a fraction of the antioxidants contained in the samples were associated to Triton X-100 micelles. A different result was obtained for orange juice and fruit pulp extracts, where no effect of micelles was observed, indicating minimal incorporation of their antioxidants into the micellar pseudophase. This result is compatible with a predominance of hydrophilic antioxidants such as ascorbic acid in orange juice and fruit extracts. 

**Table 2:** ORAC-PGR and PRAC-PGR_MIC_ values of complex mixtures ^a^.

Complex mixture	ORAC-PGR	ORAC-PGR_MIC_	ORAC-PGR / ORAC-PGR_MIC_
Red wine	36 ± 2	24 ± 3	1.5 ± 0.3
White wine	4 ± 1	3 ± 0.5	1.3 ± 0.5
Tea extract	16 ± 2	11 ± 1	1.5 ± 0.3
Seed extract of *Eugenia jambolana*	10 ± 1	8 ± 0.6	1.3 ± 0.2
Pulp extract of *Eugenia jambolana*	2 ± 0.3	2 ± 0.4	1.0 ± 0.3
Seed extract of *Myrciaria cauliflora*	15 ± 1	12 ± 2	1.3 ± 0.3
Pulp extract of *Myrciaria cauliflora*	5 ± 1	6 ± 1	0.8 ± 0.3
Orange juice	19 ± 3	21 ± 2	0.9 ± 0.2

^a^ Values represent the concentration (mM) of a Trolox solution that produces the same effect than the tested beverage.

## 4. Materials and Methods

### 4.1. Chemicals

**Figure 8:**
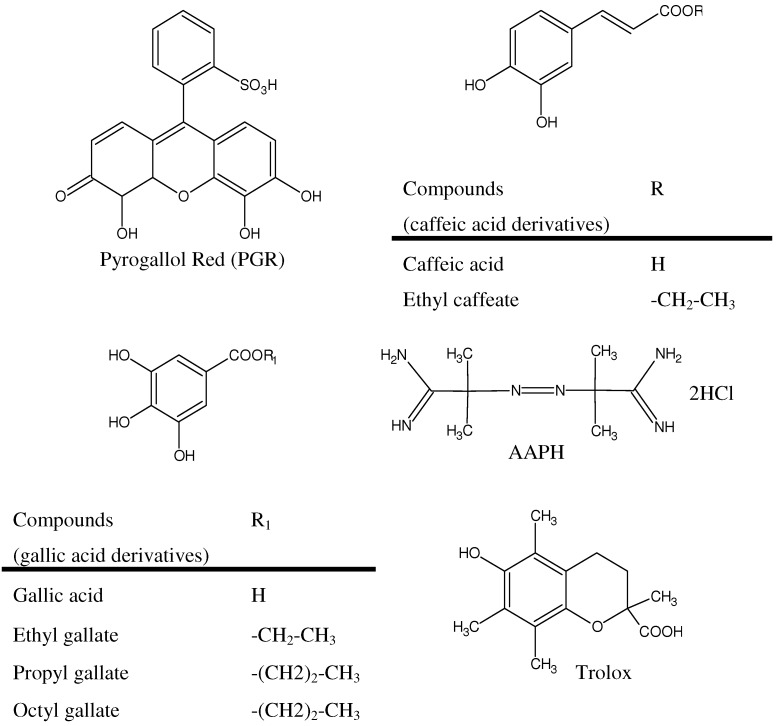
Chemical structure of pyrogallol red (PGR), Trolox, AAPH, caffeic and gallic acid derivatives.

2,2’-Azo-bis(2-amidinopropane) dihydrochloride, (AAPH), was used as peroxyl radical source. Pyrogallol red (PGR), Trolox (6-hydroxy-2,5,8-tetramethylchroman-2-carboxylic acid), all tested polyphenols (excluding ethyl caffeate), β-carotene, ascorbic acid, Triton X-100, and AAPH were purchased from Sigma-Aldrich (St. Louis, MO) and employed as received (Figure 8). 

### 4.2. Synthesis of Ethyl Caffeate

Caffeic acid (90 mg, 0.5 mmol) was refluxed in ethanol (80 mL) in presence of concentrated HCl (0.5 mL), during 8 hours. The reaction mixture was diluted with 500 mL of a saturated aqueous solution of NaHCO_3_ and extracted with three volumes of ethyl acetate (100 mL). The organic layer was dried with sodium sulfate and evaporated in vacuum to yield the ethyl caffeate (Figure 8) as a pale brown solid (76 mg ,80% yield). IR (KBr): 3436.5, 1659.0, 1282.0 cm^-1^; ^1^H-NMR (400 MHz, DMSO-d_6_)δ ppm: 9.53 (s, 1H), 9.15 (s, 1H), 7.46 (d, *J =* 15.91 Hz, 1H), 7.03 (s, 1H), 6.99 (d, *J =* 8.14 Hz, 1H), 6.75 (d, *J =* 8.14 Hz, 1H), 6.25 (d, *J =* 15.91 Hz, 1H), 4.14 (q, *J =* 7.01Hz, 2H), 1.23 (t, *J =* 7.01Hz, 3H). 

### 4.3. Sample Preparation

Polyphenols: Stock solutions of the polyphenols were prepared in ethanol immediately before their use. 

Tea infusion: Black tea bags were Chilean commercial products. Infusions were prepared by adding 150 mL of distilled water (95–100 °C) to the bags (each containing 2 g of dried tea leaves material). The infusions were brewed for 5 min. Upon withdrawing the bags, the resulting solutions were cooled to 20 °C and immediately used to assess their antioxidant properties. 

Wines: Red wine, diluted with phosphate buffer (75 mM) at pH 7.4 (1:10), or undiluted white wine were added directly to the working solution (final volume 3 mL). 

Berry extracts: Fruits of *Eugenia jambolana* and *Myrciaria cauliflora* were harvested at the commercial ripe stage. The samples were frozen and stored at −20 °C until their analysis. The extracts were obtained as follows: 20 g of each sample (seed or pulp) were homogenized in a methanol/HCl 1% (6/1, v/v) mixture. The extracts were filtered (employing Whatman N°4 filter), dried in a rotatory evaporator at 40 °C, and dissolved in methanol. The obtained concentrations of the extracts were: *Myrciaria cauliflora:* fruit pulp (38 g dried extract/L), and seed (35 g of dried extract/L). *Eugenia jambolana*: fruit pulp (25 g dried extract/L), and seed (34 g dried extract/L).

### 4.4. Working Solutions

Phosphate buffer (75 mM, pH 7.4) with or without Triton X-100 (50 mM) was employed as reaction media. A reaction mixture containing AAPH (10 mM), PGR (5 µM), and the tested sample, was incubated in the reaction media at 37 °C. PGR consumption was evaluated from the progressive absorbance decrease measured at 540 nm in the thermostatized cuvette of a Hewlett Packard 8453 (Palo Alto, CA, USA) UV-visible spectrophotometer. Control experiments were carried out employing a PGR plus AAPH solution in the absence of the tested samples. 

### 4.5. ORAC-PGR Determinations

The consumption of PGR, associated to its incubation in the presence of AAPH was estimated from absorbance (A) measurements. Values of (A/A_0_) were plotted as a function of time. Integration of the area under the curve (AUC) was performed up to a time such that (A/A_0_) reached a value of 0.2. These areas were employed to obtain ORAC values, according to Equation [1], and Equation [2], for complex mixtures and pure compounds, respectively:

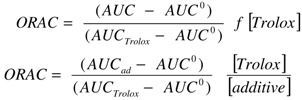

where:

AUC = Area under curve in the presence of the tested complex mixture.
AUC_ad_ = Area under curve in the presence of additives (pure antioxidants).
AUCº = Area under curve of control (PGR plus AAPH solution).
AUC_Trolox_ = Area under curve in the presence of Trolox.
f = Dilution factor, equal to the ratio between the total volume of the working solution (targetmolecules plus AAPH plus the sample aliquot) and the added sample volume.
[Trolox] and [Additive] = Molar concentration of Trolox and additive, respectively.

### 4.6. Association of PGR to Triton X-100 Micelles Assessed by Ultrafiltration

A solution (25 mL) of PGR (5 µM) in phosphate buffer (75 mM, pH 7.4) with Triton X-100 micelles (50 mM) was ultracentrifugated through a Microcom membrane (cut off = 30,000 Da). Free PGR in the ultrafiltrate was determined by UV-visible spectroscopy. All measurements were carried out at 20 ± 1 °C. Control experiments carried out in the absence of Triton X-100 micelles did not show any capture of PGR by the membrane. All experiments were carried out in triplicate.

## 5. Conclusions

Triton X-100 micelles affect the values of the ORAC-PGR index of antioxidants in a lipophilicity-dependent way. In particular, ORAC-PGR values obtained in the presence of micelles (ORAC-PGR_MIC_) decreased when the lipophilicity of the compounds increased. The ratio ORAC-PGR/ORAC-PGR_MIC_ can then be considered as a measure of the tested compound lipophilicity. This approach is sustained by results obtained employing a series of gallate derivatives and caffeic acid and ethyl caffeate. It is proposed that this approach, applied to complex mixtures, can afford a semi-quantitative estimation of the average hydrophobicity of the compounds contributing to the antioxidant activity of the tested sample.
